# Development and Experiment of an Innovative Row-Controlled Device for Residual Film Collector to Drive Autonomously along the Ridge

**DOI:** 10.3390/s23208484

**Published:** 2023-10-16

**Authors:** Zhijian Chen, Jianjun Yin, Jiaxin Yang, Maile Zhou, Xinzhong Wang, Sheikh Muhammad Farhan

**Affiliations:** School of Agricultural Engineering, Jiangsu University, Zhenjiang 212013, China; 2112116001@stmail.ujs.edu.cn (Z.C.); jiaxinyang97@163.com (J.Y.); zhoumaile@126.com (M.Z.); xzwang@ujs.edu.cn (X.W.); farhansheikh@ujs.edu.cn (S.M.F.)

**Keywords:** residual film collector, cotton stalks, cotton inspection device, kinetic models, automatic alignment

## Abstract

The field harvesting process of harvesting machinery is often affected by high workload and environmental factors that can impede/delay manual rowing, thereby leading to lower efficiency and quality in the residual film collector. To address this challenge, an automatic rowing control system using the 4mz-220d self-propelled residual film collector as the experimental carrier was proposed in this study. Cotton stalks in the ridges were chosen as the research object, and a comprehensive application of key technologies, machinery, and electronic control was used, thereby incorporating a pure tracking model as the path-tracking control method. To achieve the automatic rowing function during the field traveling process, the fuzzy control principle was implemented to adjust the forward distance within the pure tracking model dynamically, and the expected steering angle of the steering wheel was determined based on the kinematic model of the recovery machine. The MATLAB/Simulink software was utilized to simulate and analyze the proposed model, thus achieving significant improvements in the automation level of the residual film collector. The field harvesting tests showed that the average deviation of the manual rowing was 0.144 m, while the average deviation of the automatic rowing was 0.066 m. Moreover, the average lateral deviation of the automatic rowing was reduced by 0.078 m with a probability of deviation within 0.1 m of 95.71%. The research study demonstrated that the designed automatic rowing system exhibited high stability and robustness, thereby meeting the requirements of the autonomous rowing operations of residual film collectors. The results of this study can serve as a reference for future research on autonomous navigation technology in agriculture.

## 1. Introduction

The use of plastic film to cover farmland has obvious effects, such as drought prevention, warming, and moisture retention, which can effectively increase crop yield. It has been widely promoted and used in various provinces across China, most notably in regions such as Xinjiang and Inner Mongolia [1]. However, due to the nondependability of agricultural plastic film and the lack of a sound recycling mechanism, severe problems with plastic film residue have occurred, thereby leading to serious nonpoint sources of pollution such as water and soil pollution [2]. Currently, mechanical residue film recovery heavily relies on manual steering. Prolonged work can lead to high labor intensity for the driver and cannot guarantee the quality of recovery. [3]. Therefore, to meet the requirements of comprehensive green transformation for social development, it is necessary to research suitable methods for row control in the residue film recovery process with a focus on the cotton field environment to solve the problem of difficult and costly agricultural film recovery.

In recent years, there has been growing interest and significant advancements in the research and application of automatic navigation and autonomous driving technologies in agricultural machinery. These advancements have mainly been facilitated by adopting high-precision RTK satellite positioning technology for navigation. Huang et al. [4] developed a navigation system based on a combination of Bei Dou satellites and an inertial navigation system to improve the positioning accuracy and reliability of agricultural machinery vehicles. By conducting experiments in different scenarios and actual road conditions, they obtained specific position and speed information for agricultural machinery. The experimental results indicate that the positioning error of this system on open roads was within 3 cm. The heading error was within 0.6°, and it can quickly converge, even under adverse conditions. Li et al. [5] designed a fuzzy adaptive finite impulse response Kalman filter based on the combination of global navigation satellite systems and inertial measurement units to obtain the posture and position information of agricultural machinery during travel. Experimental results showed that when the speed of the tractor was 0.8 m/s under a differential GNSS state, the average error and root mean square error (RMSE) under filtering were 1.074 and 1.396 cm, respectively. Compared to unfiltered situations, this algorithm can make tracking agricultural machinery paths smoother, more stable, and more accurate, thus achieving centimeter-level tracking accuracy. However, satellite positioning technology often determines the AB line during operation. During the machine operation, the residual film collector must always be aligned with the path between the ridges where cotton was planted. Therefore, row detection and vehicle steering control are important components of the automatic row control system.

Many scholars have also completed the extraction of crop rows from a visual detection perspective, thus realizing the detection of walking paths in the field. Zhai et al. [6] proposed a multicrop row detection algorithm based on binocular vision to solve the problem of frequent weed growth in fields and image noise caused by shadows, lighting changes, irregular backgrounds, and other unexpected factors. The average absolute mean deviation angle was less than 1.05°, and the average standard deviation was less than 3.66°. Gai et al. [7] proposed an under-canopy navigation system developed using time-of-flight (ToF) cameras for extracting crop rows from depth images captured below the crop canopy. Based on the detection results, this system can generate crop field maps as grids when reliable harvester positioning was available from other sources such as GPS and IMU. In situations where field maps were available, but the positioning was unreliable, vehicle positioning data between rows could be provided. The testing results showed that the proposed system could draw crop rows of maize and sorghum fields with an average absolute error (MAE) of 3.4 cm and 3.6 cm. Yang et al. [8] addressed the issue of poor real-time performance in crop row detection under machine vision by using YOLO neural networks to extract crop row feature information and fitting the detection lines. The experimental results showed that the average error angle of the detection line was 1.88°, and processing a single image only took 25 milliseconds, which can meet the real-time and accuracy requirements of field navigation. The operating conditions in the cotton field environment can be particularly complex, because when the machinery is set in motion, factors such as dust and other environmental conditions contribute to severe background interference.

Consequently, the existing visual-based row processing methods fall short of meeting the operational requirements for rowing during field operations. Zhang et al. [9] designed an automatic row control system for corn harvesters to improve the quality of corn harvesting rows and reduce the labor intensity of drivers. This system included an automatic row-sensing system and a path-tracking control system. Field experiment results showed that the mean deviation of the automatic row control experiment based on mechanical row sensors in harvesting was 0.0876 m, the standard deviation mean was 0.0976 m, and the proportion of deviations within ±15 cm and ±30 cm was 83.1% and 100%, respectively. The results can provide theoretical support for the automatic row control of corn harvesters. To improve the automation level of cotton topping machines, Zhang et al. [10] designed an automatic rowing device for cotton topping machines that can be monitored in real time through a man–machine interface to improve driving straightness. Researchers reported that, compared with manual rowing, the average deviation of automatic rowing was reduced by 8.08 cm, a decrease of 56.62%, and the bias rate increased by 27.5%. Li et al. [11] designed an automatic row control system by integrating mechanical, hydraulic, and other key technologies for the 4UGS2 double-row potato harvester. By comparing automatic row harvesting experiments with manual row harvesting experiments, the average clear potato rate increased by 2.16%, the average damaged potato rate decreased by 1.40%, and the average missed digging rate decreased by 1.81%.

Experts have achieved partial results in designing alignment systems for agricultural machines in the field through experimental and numerical methods. However, the collision mechanism between the end sensor and the cotton pole, as well as steering characteristics during control, has not been sufficiently studied. Furthermore, with the integration of electric steering wheels, the control mechanism of self-propelled residual film collectors becomes more complex compared to conventional conditions. Therefore, there is an urgent need to determine the sensitivity of the alignment mechanism and the deviations in the steering for further research.

This research proposes a solution to the abovementioned problems by implementing an automatic alignment system for the 4mz-220d self-propelled residual film collector. The system is designed with the STM 32 controller as the main control unit, a touch-type cotton row detection device as the system input, and an electric steering wheel as the actuator. Additionally, a vehicle kinematic analysis model was established, and the pure tracking model was dynamically adjusted using fuzzy control to calculate the necessary steering angle for the electric steering wheel. The closed-loop control of the residual film collector was achieved through mechanisms such as an electric steering wheel, a hydraulic cylinder, and an angle sensor. The objective of this research is to enhance the operational efficiency of the residual film collector.

## 2. Materials and Methods

### 2.1. Working Principle and Design of Key Components

#### 2.1.1. Working Principle

**Figure 1:**
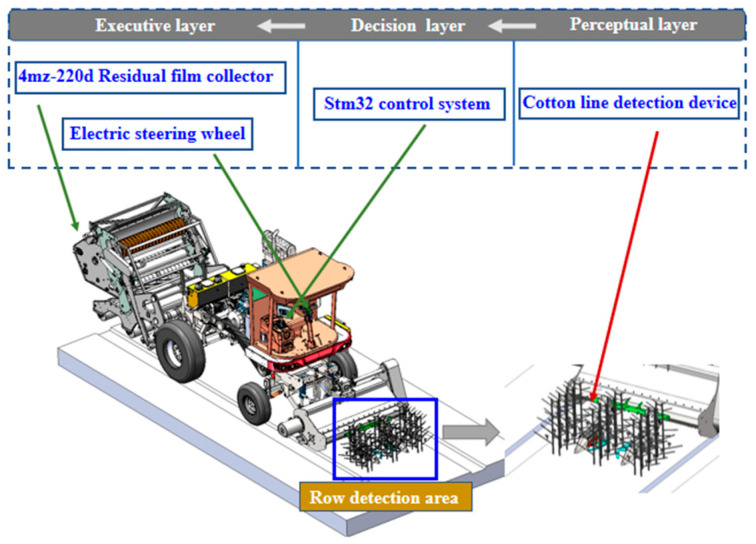
Schematic diagram of the working of the alignment system.

The automatic guidance system on the self-propelled plastic mulch retriever plays a crucial role in detecting relative lateral and heading deviations between the machine and the cotton stalks. The system consists of two key components: the touch-type cotton row detection device and the guidance control system. The mechanical guidance sensor of the touch-type cotton row detection device enables the detection of lateral deviations between the cotton stalks and the machine, while the angle sensor measures the heading deviation of the tires. An industrial computer completes the overall control of the self-propelled plastic mulch retriever. When the plastic mulch retriever is deployed to the cotton field, the mechanical guidance sensor detects the relative lateral deviation between the cotton stalks and the machine. The working principle of the system is shown in Figure 1.

The touch-type cotton row detection device was installed at the front end of the cotton stalk threshing mechanism, and an arc-shaped detection plate was installed at the front end of the device. When the automatic guidance system was started for harvesting, the arc-shaped detection plate came into contact with the cotton stalk. Based on the deviation angle of the cotton stalk detected by an angle sensor, the controller in this study output the steering angle of the electric power steering system through a fuzzy-forward distance control algorithm. This resulted in the movement of the hydraulic cylinder to adjust the front wheels, thus keeping the position of the cotton residue recovery machine in the center of the cotton row. Additionally, the front wheel’s real-time angle sensor data was utilized to determine the current heading angle of the recovery machine. By continuously analyzing the real-time signal, the controller determined whether the deviation angle had reached the required level, thus forming a closed-loop control system for automatic guidance. This control system was designed and developed to enhance the accuracy and efficiency of the cotton residue recovery process in the field.

#### 2.1.2. Cotton Growing Patterns and Characteristics

**Figure 2:**
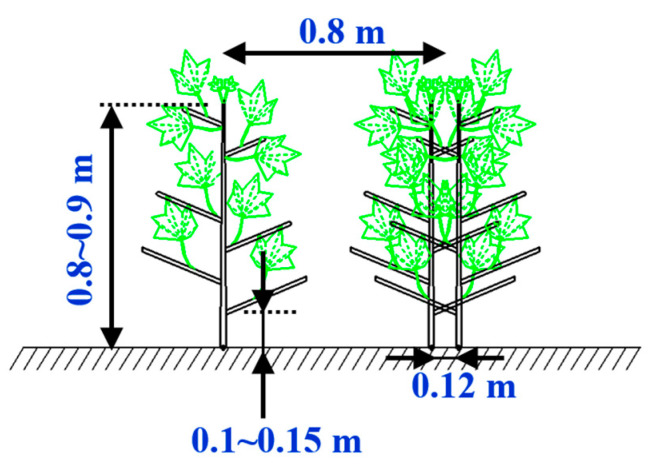
Cotton planting traits.

Compared to the previous “one row of six films,” one film of three rows was conducive to promote the growth of cotton due to the small number of acres of plants, good ventilation and light penetration, and high utilization of light and heat resources while topping; pest control was also easier than one film of six rows [12]. In the case of residual film recovery, the accuracy and efficiency of row recovery can be significantly affected if the spacing between the machine and the rower is too large or if the height is not reasonable. The average diameter of the roots of the cotton plant was measured at a height of 0.1–0.15 m from the ground, the row width and the planting width of the different rows in the field were 0.9 m and 0.12 m, respectively, as shown in Figure 2.

#### 2.1.3. Three-Dimensional Model Design of a Touch-Activated Cotton Row Detection Device

**Figure 3:**
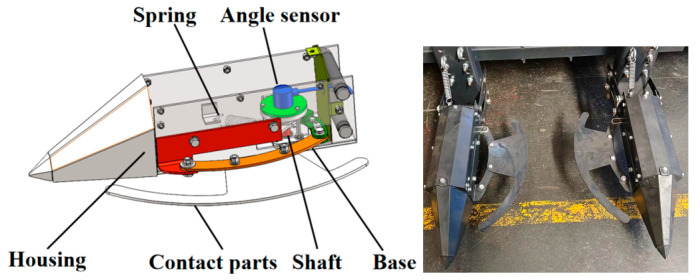
Structure of the cotton row detection device.

Mechanical alignment devices are characterized by low cost, simple construction principles, high reliability, and easy maintenance. However, traditional mechanical alignment sensors, predominantly of the travel switch type, fail to accurately detect machine offset during field operations. To address this issue, this study presents a mechanical alignment sensor tailored to the cotton field environment that calculates offset distance precisely through changes in the angle sensor output value. The touchline detection device comprises a rotating assembly and sensing assembly, with the former consisting of a rotation, shaft housing, springs, and so on, and the latter mainly consisting of an angle sensor, as presented in the 3D model in Figure 3.

**Figure 4:**
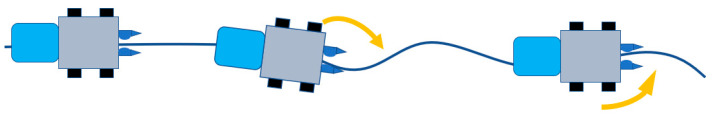
Diagram of the institutional role model.

The principle of operation of the touch-activated cotton row detection device on cotton stalks is shown in Figure 4. This device operated in three different modes: (1) neither side of the cotton stalk was in contact with the device, (2) the left side of the cotton stalk was in contact with the device, and (3) the right side of the cotton stalk was in contact with the device. When the lower side of the cotton plant was deflected on the main stalk side, the corresponding detecting mechanism was activated, thus resulting in the oscillation of the detecting rod and the rotation of the spindle and angle sensor, which then transformed the lateral deflection of the cotton plant into an angular change; upon passing the cotton plant, the torsion spring causes the detecting rod to return to its initial position.

#### 2.1.4. Adams-Based Simulation of the Motion of a Cotton Row Detection Device

**Figure 5:**
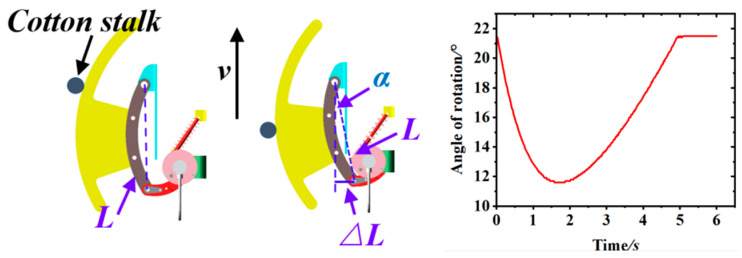
Simulation results of Adams.

To comprehensively investigate the operational characteristics of the touchline inspection device, a 3D solid model was created using advanced design software and subsequently exported to X_T format. The dynamic simulation software Adams2020 further simulated the detection mechanism’s trajectory to enhance our understanding of the device’s functionality [13]. In the simulation environment, the cotton plant was arranged such that the contact part gradually formed a larger angle with the forward direction upon contact with the plant. This continued until the torsion spring torque caused the detection rod to reset after completely passing the cotton plant. As a result, the curve representing the angle change with respect to the forward direction exhibited a small value point after the detection had passed the cotton plant. The calculation formula for determining the forward direction offset is expressed as follows:

(1) ΔL=Lcosα

where *α* is the deflection angle, the lateral deflection, and *L* is the distance between the two pivot points of the contact arm. The simulation results were obtained through Adams software, shown in Figure 5, and the lateral offset can be obtained by analyzing the extreme points of the analog signal change curve generated by the angle sensor.

### 2.2. Kinetic Model for Residual Film Collector

#### 2.2.1. Vehicle Steering Models

**Figure 6:**
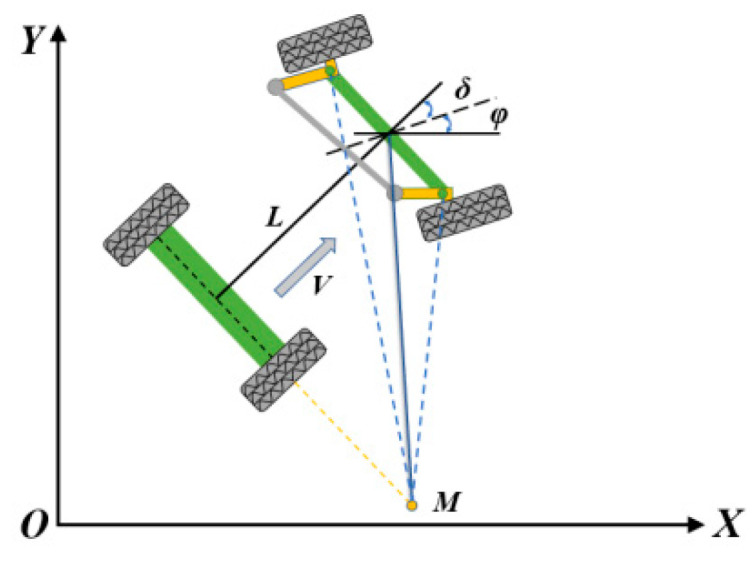
Steering model of the residual film collector.

The traveling mechanism of the self-propelled residual film collector was based on the Ackermann steering principle [14]. In this research, it was assumed that there would be no lateral force during the driving process of a wheeled vehicle with front-wheel steering and rear-wheel drive. Moreover, the motion of the front wheels was considered pure rolling, without considering the influence of the material of the tires, as well as other factors. In this way, a dynamic steering model [15] was established for the steering of the recycling machine, as shown in Figure 6.

By simplifying the vehicle to a two-wheeled bicycle model in the inertial coordinate system *XOY*, the following equation can be obtained based on the geometric relationships.

(2) $$\left[\begin{array}{c} \dot{x}\\ \dot{y}\\ \dot{\varphi } \end{array}\right]=\left[\begin{array}{c} \cos(\varphi )\\ \sin(\varphi )\\ \tan(\delta )/L \end{array}\right]v$$

In this case, *x*, *y*, *δ*, *φ*, *L*, and *v* are the horizontal coordinates, vertical coordinates, front wheel angle, heading angle, axis distance, and speed, respectively. An electric motor drove the steering wheel of the self-propelled residual film collector, and the whole autosteering system can be regarded as a first-order linear kinematic model [16] as expressed in the following expression:

(3) $$\dot{\delta }=-\frac{1}{T}\left(\delta -\delta_{U}\right)$$

where $\delta_{U}$ is the virtual front wheel target steering angle of the recovery machine, and *T* is the steering system time constant.

According to the Ackermann geometry model, the steering curvature was related to the front wheel steering angle *δ* by the following equation:

(4) $$\gamma =\frac{\tan\delta }{L}$$

where *γ* is the actual steering curvature of the residual film collector.

Assuming that the instantaneous tracking path of the vehicle is straight and the front wheel turning angle is small, Equation (3) can be linearized as follows:

(5) $$\gamma =\frac{\delta }{L}$$

The process of changing the steering curvature of a vehicle can therefore be seen as a first order Markov process.

(6) $$\dot{\gamma }=-\frac{1}{T}\left(\gamma -\gamma_{U}\right)$$

where *γ_U_* is the target steering curvature of the tractor.

According to Equations (2), (4) and (6), the kinematic model of the recovery machine can be obtained as follows:

(7) $$\left\{ \begin{array}{l} \dot{x}=v\cos\varphi \\ \dot{\varphi }=v\gamma \\ \dot{\gamma }=-\frac{1}{T}\left(\gamma -\gamma_{u}\right) \end{array}\right.$$

#### 2.2.2. Pure Pursuit Algorithm

**Figure 7:**
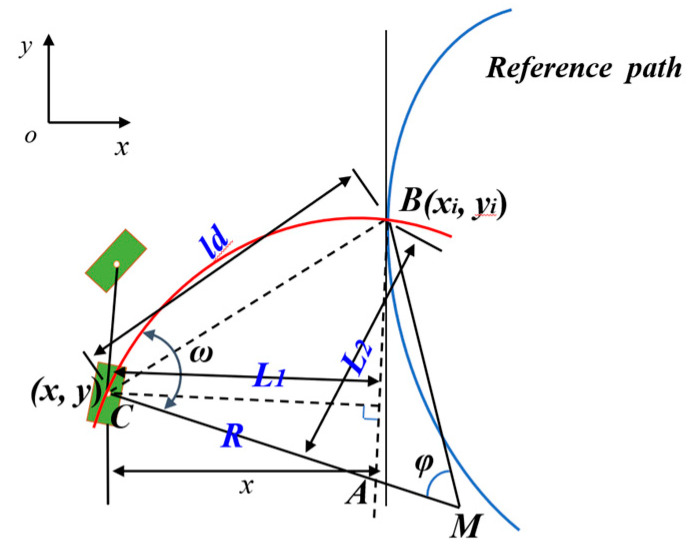
Pure pursuit algorithm.

The pure tracking model is a geometric tracking model [17], as shown in Figure 7. In the diagram, *M* is the instantaneous center of the circle when the vehicle is turning, and when the forward-looking distance *l_d_* is determined, a unique point along the desired path can be found as a presighting position. A circular arc trajectory was determined based on the vehicle’s rear axle center position coordinates, the time heading angle, and the presighted location coordinates. The radius of the arc trajectory was then calculated, and the vehicle control volume was derived to achieve path tracking. As can be seen from the diagram, *l_d_* is the variable to be determined, where (*xi*, *yi*) are the coordinates of the target point.

(8) $$\{\begin{array}{c} L_{1}=-R(1-\cos\omega )\\ L_{2}=-R\sin\omega \end{array}$$

*R* is the instantaneous radius of turn; *A* is the intersection of a line parallel to heading *MB* and radius *MC*; *B* is the target point; *C* is the center of the rear axle; *ω* is the angle of circumference corresponding to chord BC; *L*_1_ is the distance from point *A* to point *B*; *L*_2_ is the distance from point *A* to point *C*; and *l_d_* is the forward distance. From the geometrical relations, it follows that

(9) $$L_{1}{}^{2}+L_{2}{}^{2}=l_{d}^{2}$$

(10) $$L_{1}=-x\cos\left(\varphi -\frac{\pi }{2}\right)+\sqrt{l_{d}^{2}-x^{2}}\sin\left(\varphi -\frac{\pi }{2}\right)$$

(11) $$R=\frac{L}{\tan\delta_{U}}$$

When combining Equations (8) and (9), that the following is obtained:

(12) $$2R\times L_{1}=l_{d}^{2}$$

Substituting into Equations (10) and (11) into (12) yields the following:

(13) $$\delta_{U}=\arctan\frac{2L\left(x\sin\varphi +\sqrt{l_{d}^{2}-x^{2}}\cos\varphi \right)}{l_{d}^{2}}$$

The curvature was calculated as in Equation (14):

(14) $$\gamma_{U}=\frac{\tan\delta_{U}}{L}=\frac{2\left(x\sin\varphi +\sqrt{l_{d}^{2}-x^{2}}\cos\varphi \right)}{l_{d}^{2}}$$

### 2.3. Simulation of Control Strategies Based on MATLAB/Simulink

To verify the feasibility of the designed automatic alignment control system, it was simulated using MATLAB/Simulink2022 software before mounting it on a real machine for testing. Traditional control methods include PID control, optimal control, neural network control, etc. However, these methods have certain limitations in the application of agricultural machinery, such as difficulty in determining the parameters of PID control, poor adaptability of optimal control to nonlinear agricultural machinery models, and complex neural network algorithms. Fuzzy control can be described in simple language to solve complex control problems in engineering. By expressing control laws through expert experience, the closer the expert experience is to the real motion law, the greater the control effect will be. Therefore, for multiple-input, single-output systems, fuzzy control is suitable [18]. 

Its key components include domain partitioning, affiliation, and fuzziness [19]. The lateral deviation *Te* and the heading deviation *θe* were the inputs to the fuzzy controller; the range of the heading deviation was [−15, 15] in °, and the range of the lateral deviation was [−0.2, 0.2] in *m*. Fuzzy quantities were obtained by fructifying both, and, finally, the forward-looking distance *l_d_* was obtained by inferring and fructifying the rules. The negative sign in the range only represents the direction of a numerical value, not a negative number. The state of the input and output quantity is represented by “large, medium, and small”, and there are positive, negative, and zero states. Therefore, the fuzzy set of each variable can be divided into seven levels: [negative big, negative medium, negative small, zero, positive small, positive medium, positive big]. Therefore, the fuzzy set is represented as [NB NM NS ZO PS PM PB]. Therefore, the system inputs *Te* and *θe* have fuzzy subsets [NB NM NS ZO PS PM PB], and the output variable *l_d_* has a fuzzy subset [NB NM NS ZO PS PM PB]. 

Related studies have shown that the forward-looking distance was closely associated with human driving behavior when the distance traveled by the vehicle was 2 to 3 s [20]. The basic domain of the forward-looking distance was therefore selected as 2 to 8 in *m*. The fuzzy controller determined the appropriate value of *l_d_* according to the different values of *Te* and *θe* to achieve optimal control performance. The input and output variables were governed by specific control rules, namely, these included the following: in the event of a large lateral deviation, the priority was to minimize the deviation and reduce the lateral deviation as soon as possible; in the case of a small lateral deviation, emphasis was placed on preventing overshoot and maintaining system stability. These principles led to the formulation of 49 control rules, which, when combined, resulted in a fuzzy control rule table illustrated in Table 1.

**Figure 8:**
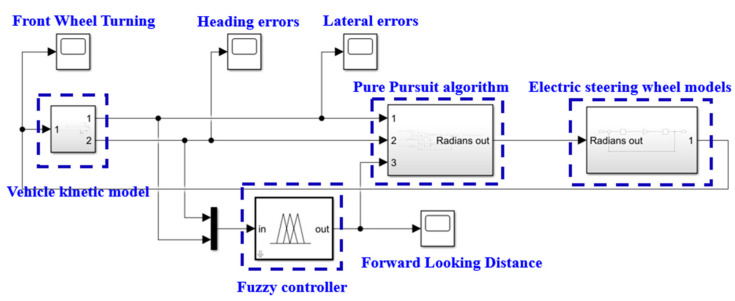
Simulation model of the residual film collector.

To compare the operational performance of the residual film collector under different parameters, this part of the study presented a simulation model for automatic alignment control in agricultural harvesters, as shown in Figure 8. The model took inputs of lateral deviation and heading deviation angle θ detected by an automatic alignment sensing system and produced the forward-looking distance using fuzzy control rules. The output of the pure tracking model was for calculating the adjustment angle of the front wheels of the harvester steering, which was then passed to the kinematic model of the residual film collector after the electric steering wheel. The rate of change of the deviation angle was obtained, and the real-time lateral deviation and heading deviation were determined according to the dynamic differential equation. The alliterative model applied these calculations until the lateral deviation and heading deviation were close to zero.

### 2.4. Automatic Alignment Control System Design

#### 2.4.1. Selection of Sensors

**Figure 9:**
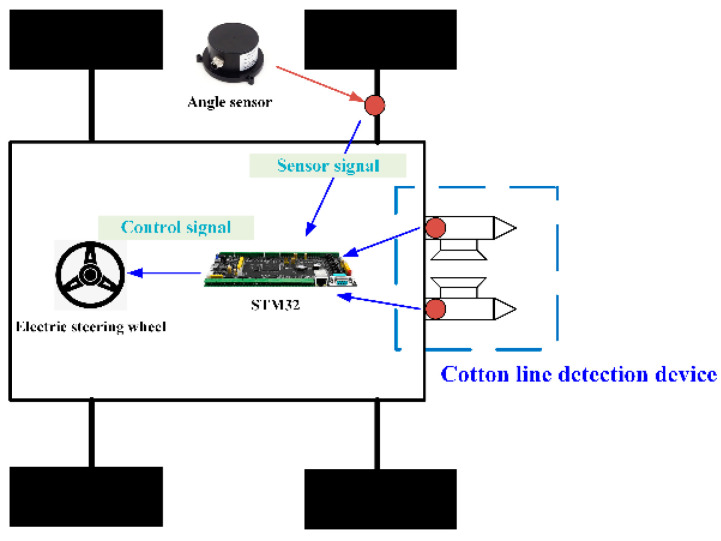
Hardware structure of the control system.

The harsh and complex harvesting environment in the farmland requires robust sealing, antivibration properties, and high-precision lateral deviation detection. The P2020 high-precision magnetic angle sensor from Mirante, capable of being integrated with various types of computers and collectors, was used for lateral deviation detection. The environment around the field was complex and changing, and the system was designed to ensure that the steering wheel could work with high accuracy and safety while also to make it versatile and have both manual and automatic driving modes, which can be achieved with an electric steering wheel. The 175ACMS electric steering wheel from Shanghai Lianshi was chosen for its ability to drive the direction directly, while the controller utilized a set control algorithm to calculate motor direction, speed, and angle for automatic steering based on navigation information received on the CAN bus. The selected line system was based on the ARM series STM32 processor, receiving real-time data access through ports, as shown in Figure 9. The control system was powered by two modules, with a 5 V control signal voltage for the sensor, microcontroller, and motor driver, and a 24 V electric steering wheel drive voltage.

#### 2.4.2. Software Architecture of the Control Program

**Figure 10:**
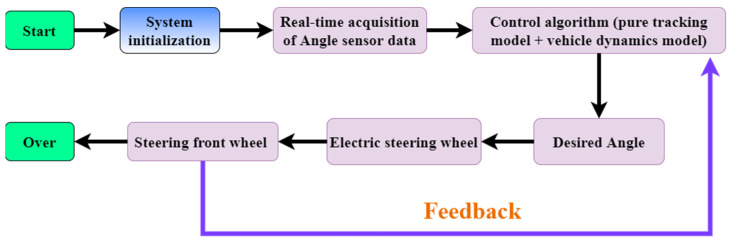
Control system structure.

After the integration of the data information, the alignment system was developed, downloaded, and debugged using the Keil V5.14 development software for the control program [21]. The whole control system was based on the end-to-end control principle, where the STM32 development board acquired sensor data from the touch cotton line detection device, processed it, and transmitted the steering signal to the electric steering wheel to achieve controlled movement of the electric steering wheel. The angle sensor installed on the left front wheel provided real-time information on the vehicle’s heading deflection angle. Upon powering up, the system automatically initialized the STM32 control system by operating the cotton row detection mechanism to adjust the cotton rod rows to be centered. The main program collected the deflection angle of the detection mechanism, and based on the judgment of the left and right sensors, the steering wheel turned right or left. If neither sensor triggered, the steering wheel remained unmoved. The steering wheel control algorithm combined a pure tracking model with a vehicle kinematic model to generate the desired angle for the steering wheel, and the actual angle was fed back to the algorithm to complete the closed-loop control of the system. To illustrate the control system, a diagram is presented in Figure 10.

### 2.5. Field Experiments

**Figure 11:**
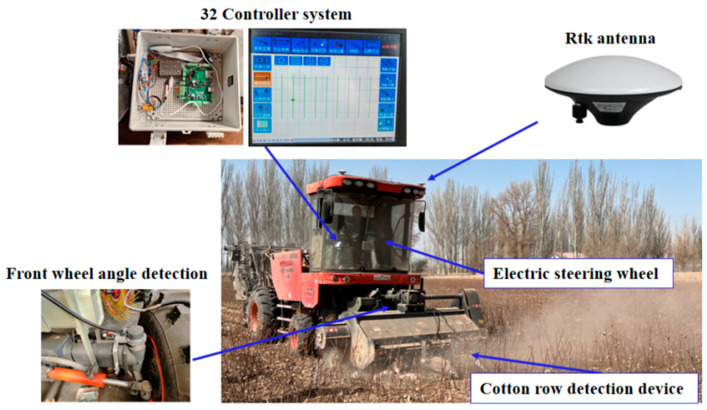
Field experiment of the residual film collector.

To further verify the performance, reliability, and control accuracy of the autonomous residual film collector control system proposed in this research, a series of field experiments were conducted on the 4mz-220d self-propelled residual film collector jointly developed by Jiangsu University, Shihezi University, and Changzhou Hanson Machinery Company. The experiments were conducted at a farm experiment base of the 50th Regiment in Tumxuk City, Xinjiang, China, with a variety of environmental conditions, including sandy soil, rough road conditions, and a temperature of 14℃, as shown in Figure 11. The objective of the experiments was to ensure that the movement trajectory of the retrieval machine matched the position of the cotton stalk for precise film retrieval. Therefore, the vehicle’s motion trajectory was recorded during the experiment and compared with the cotton stalk plant trajectory after the experiment to determine the alignment under different conditions. The Beidou dual antenna RTK satellite positioning system was used to achieve the positioning and orientation of the residual film recycling machine. The system supports dual antenna input, with a positioning accuracy of 0.01 m and a directional accuracy of 0.08° in RTK mode. To function, determine the starting and ending points of the expected path for path tracking through a satellite positioning system (GPS), and store longitude and latitude data during walking. To calculate the actual deviation of the machine’s trajectory during the experiment, the Beidou satellite system was used for coordinate transformation and other processing. The detection value was compared with the actual value to verify the accuracy of the film retrieval system.

## 3. Results and Discussion

### 3.1. Analysis of Simulation Data

**Figure 12:**
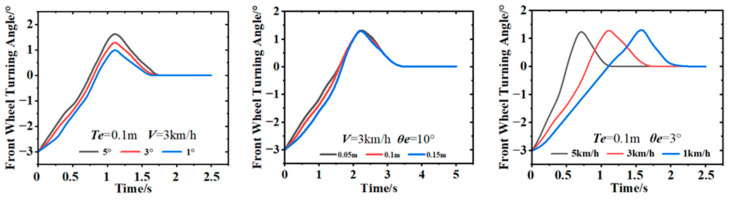
Simulation results of MATLAB/Simulink.

The above simulation model conducted simulation analysis based on the initial conditions. The simulation time was 10 s, and all other parameters were set at default values. The wheelbase of the residue-recalling machine was 2.9 m. Different heading deviations, speeds, and lateral deviations were given, and the simulation results are shown in Figure 12.

**Figure 13:**
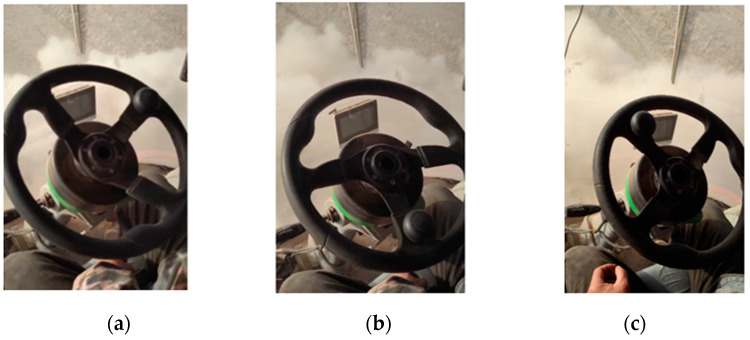
Electric steering wheel deflection: (**a**) left deflection, (**b**) normal walking, and (**c**) right deflection.

When the heading and lateral deviation were fixed, the response speed of the system gradually accelerated. As the speed of the vehicle increased, the front wheel turning angle exhibited fluctuations during the steering process, thereby progressively decreasing before approaching stability. When the vehicle speed and lateral deviation were stabilized, the variations in the front wheel’s turning angle under different lateral deviations were not significant. It gradually converged to the same value with time. The smooth changes in the turning angle prevented violent oscillations during path tracking. Meanwhile, when the speed and lateral deviation were held constant, the response of the front wheel’s turning angle became more pronounced with differing heading angles. This turning angle decreased gradually until the path was tracked; the process effectively suppressed the increase in deviation. Based on the simulation results, it is evident that during the whole process of walking, the steering wheel of the touch cotton line detection device quickly responded to deflection adjustment with a short response time; at the same time, the system, according to the signal of the steering wheel angle detection device, can make judgments and control the body back to the right based on the signal from the steering wheel angle detection device, without exhibiting any significant oscillation. Therefore, it can be seen that the automatic alignment system demonstrated excellent tracking effectiveness with stable performance and could carry out the next experiments, as shown in Figure 13.

### 3.2. Analysis of Field Experiment Data

**Figure 14:**
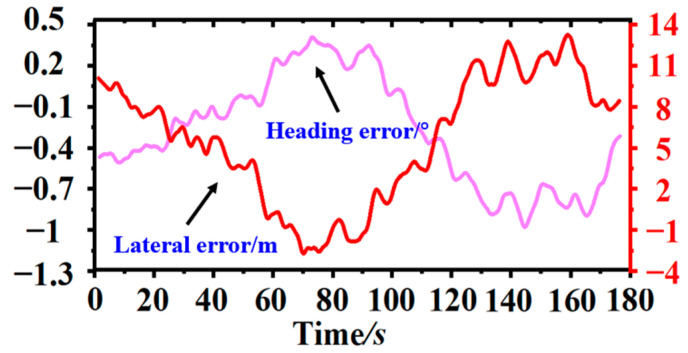
Results of manual alignment path and deviation results.

Firstly, the driver manually controlled the mulch harvester at dynamic speed, and the cotton field was sectioned and marked at a 50 m distance before the experiment, thereby keeping a 5 m distance before and after each section as the acceleration and deceleration distance of the mulch harvester. A GPS was mounted on the mulch collector to detect the movement of the harvester in real time, and the coordinates of the cotton stems were measured with the GPS in hand after the operation was completed. The GPS sensor data were collected at a frequency of 10Hz/s and printed to a file. When driven manually, the machine was able to carry out the film recovery operation, and the results of the movement trajectory and deviations were calculated and converted for the row driving effect under manual control. To ensure the effectiveness of the operation, vehicles may constantly deviate and move in opposite directions during the moving process. Therefore, this article mainly analyzed the effects of the row control, as shown in Figure 14.

**Figure 15:**
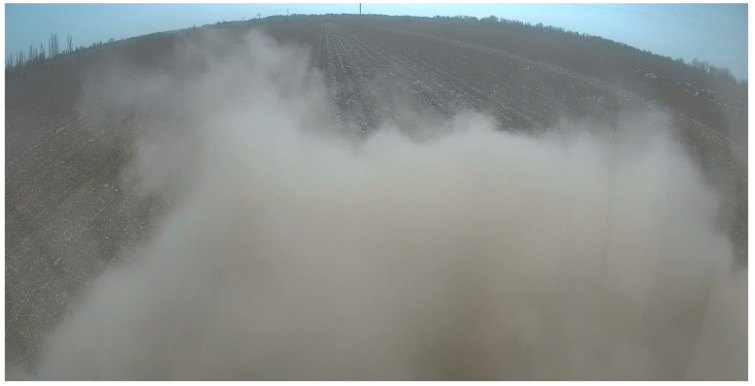
Dust masking scenario during manual alignment.

The main reason for the significant deviation in manual alignment was that the residual film collector generated a large amount of dust during operation. The operation of the driving recorder installed in the vehicle is shown in Figure 15. At this time, the operator’s front view was obstructed and could only rely on driving experience to operate the machine effectively, thereby making it difficult to perform precise manual alignment operations, thus resulting in significant deviations during walking.

**Figure 16:**
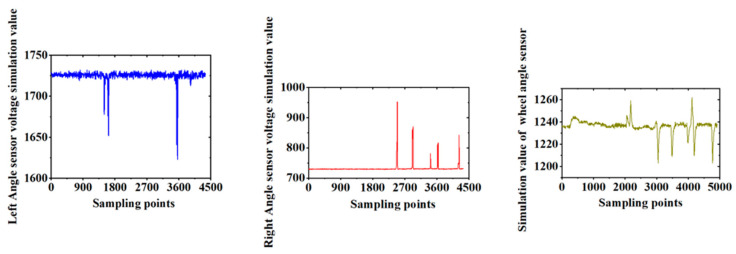
Information of simulated values.

When the residual film collector entered the cotton field to start the film retrieval operation, the mechanical alignment sensor installed on the beating rod mechanism detected the position of the cotton stalk and performed an automatic alignment experiment. The stability of the electric power steering system was crucial to the overall stability of the autonomous driving system. To adjust the electric power steering speed, it was necessary to modify the controller’s execution program and set the electric power steering to a specific speed on the upper computer. The response effect of the steering wheel was observed through an angle sensor. To ensure the reliability of the automatic alignment system, an experiment was conducted using a wooden rod to simulate the cotton stalk. The control model and actuator were tested by analyzing the experiment results. Finally, the system was used for field experiments, and the variation in the left-angle sensor offset angle, the right-angle sensor offset angle, and the vehicle’s front wheel angle measurements are shown in Figure 16.

**Figure 17:**
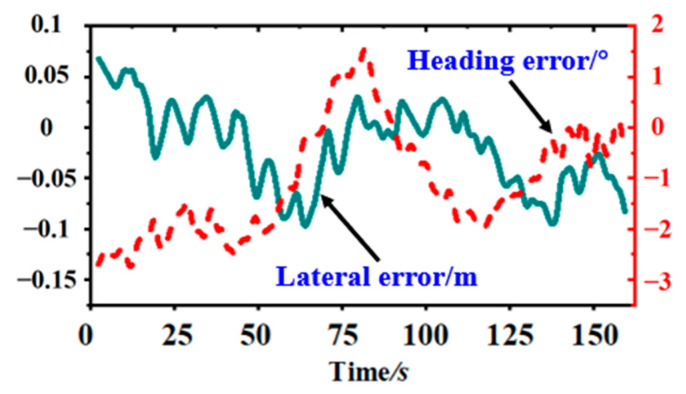
Result of automatic alignment deviation results.

During the field experiments, only the harvester’s throttle pusher was controlled to adjust the forward speed to the required value, after which the automatic alignment control system adjusted the harvester’s forward direction. The result of the deviation experiment at a forward speed of 4.6 km/h, as shown in Figure 17., was significantly better than those obtained through manual driving.

The results are presented in Table 2, and it can be observed from the above experiment results that as the forward speed of the recovery machine increased, the mean deviation between the track and the cotton rows tended to increase. This was primarily caused by the fact that, with relatively constant detection and actuator delay times, higher forward speeds resulted in fewer adjustments by the harvester over the same forward distance, thereby leading to a more significant deviation from the rows. The average deviation of the automatic row alignment was 0.066 m, and the average deviation within ± 0.1 m was 95.7%.

## 4. Conclusions

This paper investigated the control system under mechanical alignment touch characteristics using a combination of mechanical alignment and an electric steering wheel. A 4mz-220d self-propelled residual film collector was utilized as the experimental platform for testing the steering travel control performance. The findings are as follows:

(1) In view of the current situation that the residual film collector mainly relies on manual alignment, an automatic alignment system for the residual film collector based on the combination of a touch control mechanism and an electric steering wheel has been established, and the alignment experiment results demonstrated good path matching with the cotton pole, thereby laying a solid foundation for the development of alignment in the residual film collector.
(2) Based on kinematic models of the touch detection mechanism and the body steering control system, an automatic alignment control system using fuzzy adaptive control was designed and simulated in MATLAB/Simulink for deviation correction under different parameters.
(3) The average deviation of the automatic row alignment was 0.066 m, and the average deviation was within ± 0.1 m, respectively. During the automatic row alignment harvesting process, the deviation of the automatic row alignment gradually increased with increasing harvester speed. To eliminate the speed limitation, further research will focus on developing innovative control strategies to reduce the deviation in the system at high-speed operation.
(4) This study lays the foundation for the automatic running of the residual film collector, but due to limitations in equipment time and other conditions, the braking, speed regulation, and automatic path tracking in the automatic operation system of the residual film collector still need further improvement. For example, there is a close relationship between the effectiveness of the automatic alignment operations and the field environment, and further research is needed on the interaction between the residual film recycling machines, soil, and cotton stems; In addition, the field environment was harsh, and the machine vibration was obvious. Relevant protective devices can be designed to improve the stability of the sensing device, thus ultimately achieving the goal of optimizing the control system. In future research, we will continue to research new row-controlled devices and control strategies to improve the operational efficiency of the residual film collector.

## Figures and Tables

**Table 1:** Fuzzy control rules.

	*ld*	*Te*						
*θe*		*NB*	*NM*	*NS*	*ZO*	*PS*	*PM*	*PB*
*NB*		*NB*	*NB*	*NB*	*NB*	*NM*	*NM*	*NB*
*NM*		*NB*	*NB*	*NM*	*NM*	*NS*	*NS*	*NB*
*NS*		*NB*	*NM*	*NM*	*NS*	*ZO*	*NS*	*NM*
*ZO*		*NM*	*NM*	*NS*	*ZO*	*PS*	*ZO*	*NM*
*PS*		*NM*	*NS*	*ZO*	*PS*	*PS*	*PM*	*NS*
*PM*		*NS*	*ZO*	*PS*	*PS*	*PM*	*PM*	*ZO*
*PB*		*ZO*	*PS*	*PM*	*PM*	*PM*	*PB*	*PM*

**Table 2:** Results of pairwise experiments at different speeds.

Number	Forward Speed (m/s)	Average Deviation/m		Automatic Alignment of Line Track and Cotton Line Deviation
		Manual Alignment	Automatic Alignment	Percentage of Deviations within ±0.1 m (%)
1	0.64	0.083	0.026	98.6
2	0.64	0.095	0.031	97.8
3	0.86	0.115	0.052	96.3
4	0.86	0.132	0.064	97.4
5	1.17	0.168	0.077	94.7
6	1.17	0.174	0.085	95.3
7	1.37	0.186	0.094	93.7
8	1.37	0.204	0.097	91.9
Average		0.144	0.066	95.7

## Data Availability

All of the data generated or analyzed during this study are included in this published article.
